# AlSi10Mg in Powder Bed Fusion with Laser Beam: An Old and Boring Material?

**DOI:** 10.3390/ma15165651

**Published:** 2022-08-17

**Authors:** Michael Rasch, Dominic Bartels, Shoujin Sun, Michael Schmidt

**Affiliations:** 1 Institute of Photonic Technologies, Friedrich-Alexander-Universität Erlangen-Nürnberg, 91052 Erlangen, Germany; 2 Collaborative Research Center CRC 814—Additive Manufacturing, 91052 Erlangen, Germany; 3 Erlangen Graduate School in Advanced Optical Technologies (SAOT), Friedrich-Alexander Universität Erlangen-Nürnberg, 91052 Erlangen, Germany; 4 School of Engineering and Built Environment, Griffith University, Gold Coast, QLD 4111, Australia

**Keywords:** PBF-LB/M, AlSi10Mg, tailored microstructure, direct aging, microhardness, Gaussian, Top-Hat

## Abstract

Powder bed fusion with laser beam of metals (PBF-LB/M) is a widely used technology to produce parts with a high freedom in design paired with excellent mechanical properties. The casting alloy AlSi10Mg features a wide process window and a microstructure with excellent mechanical properties which are not obtainable through conventional manufacturing. One possibility for achieving this is by influencing the solidification which then directly affects the local part properties. In this study, the effect of different laser beam profiles with gaussian and top-hat intensity distributions, as well as the influence of varying parameter sets on the microstructure and microhardness within the same specimen, was examined. A test specimen consisting of many small cubes was built with different parameters. It was found that the local properties can be varied in a wide range. Build-height-dependent in-situ aging effects can thereby be exploited for tailoring the local material properties. Thus, an extra degree of freedom is added to the design of additively manufactured parts.

## 1. Introduction

The fields of application for additive manufacturing (AM) are rapidly growing. The steady development and optimization of the process itself leads to a huge potential for the fabrication of 3D parts with integrated functionalities and outstanding properties [1]. Powder bed fusion with laser beam of metals (PBF-LB/M) offers the opportunity to design highly complex parts of almost any shape [2]. 

Over time, after some failures with sintering experiments of multi-material powders [3], the PBF-LB/M process started to establish. First, metals with a low thermal conductivity like 316l [4] and Ti-Al6-V4 [5] were subject to intensive research and became available for industry. Finally, some years later, the first aluminum specimens were built successfully [6]. Improvements of the processing, the implementation, and correct use of pre-heating [7], as well using dried powder [8], enabled the production of fully dense large parts in a wide process parameter window. Hereby, it was found that the resulting microhardness strongly depends on the parameters used [7].

Since then, Aluminum alloys, especially cast alloys like AlSi10Mg [9], AlSi12 [10], or AlSi9Cu3 [11], have been the topic of several publications. Most interestingly, all these alloys are characterized by a much higher yield strength (YS) in the as-built condition compared to their conventionally produced counterparts in the untreated or heat-treated condition. This phenomenon is caused by an effect that could not be observed in casting applications before. Due to the rapid cooling during PBF-LB/M, the solidification does not take place in the equilibrium state but rather through the segregation of the alloying elements, thus causing a grain structure with almost pure aluminum in the grain center and a thin silicon shell [12]. Nevertheless, this strengthening silicon network can be dissolved due to post or in-situ heat treatment at temperatures above 294 °C [13]. With rising temperature and time, these primary participations dissolve. At first, many small flake-like silicon particles precipitate, which then become less numerous but bigger in size [14]. As a result, the initial high YS is reduced and the parts with lower YS but higher elongation at break (A) are formed. In contrast to the heat treatment of parts produced via casting, the time and temperature of subsequent artificial aging depends on the parameters used for solution annealing. In this manner, the desired Mg_2_Si-precipitations, which are responsible for Orowan hardening, can be formed [15].

The aim of the study is to investigate whether the use of different parameter sets in PBF-LB/M allows for achieving different solidification conditions. Through this adjusted parameter sets, the goal is to obtain a specimen with locally defined mechanical properties. To do so, we will take advantage of and exploit the large process regime of AlSi10Mg. The possibility to add a tailored microstructure to a part in PBF-LB/M would open a new lever and designing highly sophisticated products.

## 2. Materials and Methods

### 2.1. Machines and Materials

**Figure 1:**
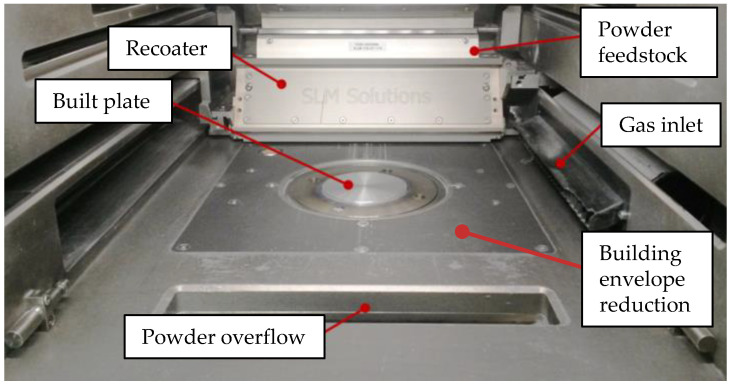
SLM 280^HL^ with installed building envelope reduction.

The experiments were conducted on a PBF-LB/M machine SLM 280^HL^ of SLM Solution Group AG (SLM) Lübeck, Germany. It is equipped with a single mode (SM) fiber laser and a multimode laser. The SM fiber laser provides a maximum laser power (P_L,max_) of 400 W at a wavelength of λ = 1070 nm and a spot diameter d_L_ of 78 μm. The multimode laser provides P_L,max_ = 1000 W at the same wavelength but d_L_ = 650 μm. Argon was used as shielding gas. A customized building envelope reduction was used in order to keep the needed powder batch size small (see Figure 1). The temperature T_sens_ of the built plate was measured directly below a 15 mm thick mounting adapter on that the substrate plate with a thickness of 5 mm and a diameter of 90 mm was screwed. Both are made of EN AW-5083. During the built job, the preheating was turned off. The residual oxygen was below 0.1% at the start of the built job and was below the detection limit after 6 h. 

### 2.2. Used Material

**Figure 2:**
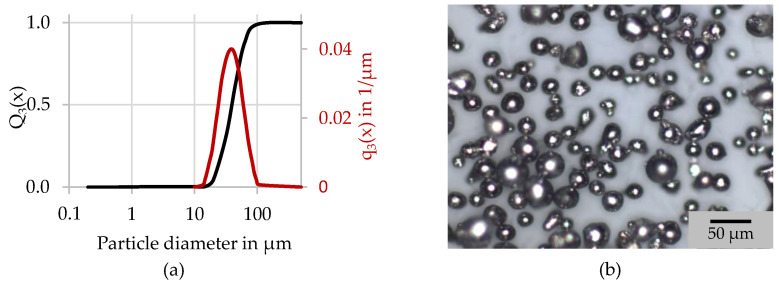
(**a**) Particle size distribution, (**b**) light microscope image of the particle form [16].

The powder used for this study was bought from SLM. The presented characteristics are taken from the analysis certificate provided by SLM (see Table 1 and Figure 2) [16]. 

The chemical composition is well in range of the allowed specification according to DIN EN 1706 [17]. The particle size was determined on the powder dispersed in isopropanol via laser diffraction using a PSA form Cilas (see Figure 2a). The particle form was found to be close to spherical (see Figure 2b). Likewise, it can be seen that only a small number of satellites are attached to bigger particles. 

### 2.3. Used Process Parameters

**Figure 3:**
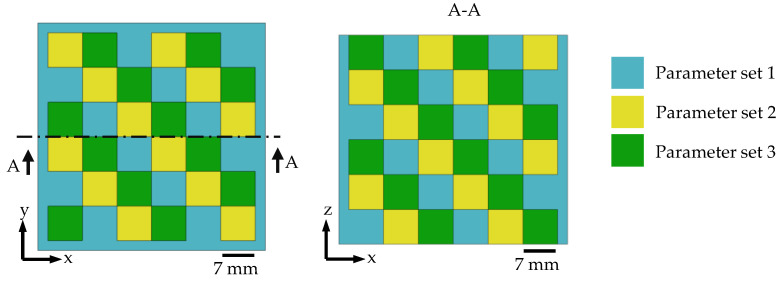
Arrangement of the parameters within the large cube.

For the experiment, one large cube was built, consisting of 6 × 6 × 6 (X × Y × Z) small cubes with an edge length of 7 mm and a 2 mm contour surrounding the arrangement of small cubes (total size 46 × 46 × 42 mm^3^). Three different types of parameter sets (PS) (see Table 2) were used so that *PS* 1–3 are alternating in X, Y, and Z directions (see Figure 3). The layer thickness of 30 µm and the scan vector rotation of 37° were kept constant for all 3 *PS*. 

PS 1 was chosen as standard PBF-LB/M-AlSi10Mg parameter for this layer thickness (30 µm). A fine dendritic grain structure with a well-defined silicon network [12] is expected. Knowing that the use of a large Top-Hat profile in PS 3 requires a contour, this PS 1 was chosen for the outline contour. PS 2 instead is the PS with the highest v_s_ currently possible (due to aging effects the maximum P_L_ of 400 W is not reached), resulting in fully dense specimens. The high scanning speed directly influences the cooling rates and therefore the microstructure that is supposed to be similar but much finer than with PS 3 [18]. PS 3 makes use of a second laser source. The scanning speed is much lower than in PS 1 and 2, whereas d_L_ is almost one magnitude larger. Both circumstances are supposed to lead to a much lower cooling rate and to locally longer exposure. The microstructure is expected to be coarser [18]. The silicon network might already be degenerated through the longer heat impact [13]. 

### 2.4. Characterization

**Figure 4:**
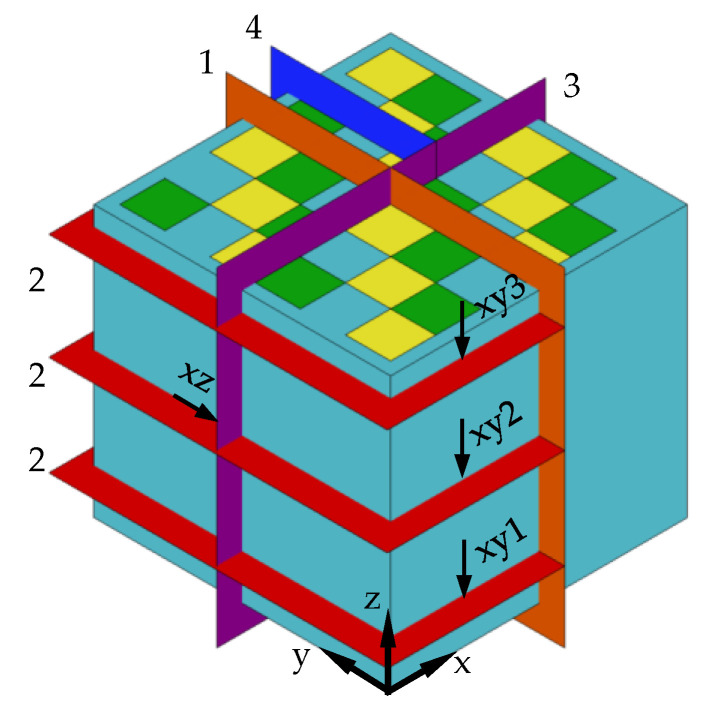
Arrangement of the cuts for the characterization with the indication of the view direction on curtain surfaces.

The final cube was first cut in the middle in z-direction (see Figure 4 plane 1). Then one half was cut again into 3 slices in a distance of 7, 21, and 35 mm, respectively, from the baseplate (see Figure 4 plane 2). The cross-sections were grinded (P240–P1200) and polished with 6 µm diamond suspension, followed by a 0.05 µm OP-S finish. Polishing was done manually in all cases. The microhardness, HV_0.1_, was determined on the surfaces of the cross-section marked with xz and xy1–3 (see Figure 4) with a 1 mm grid with the microhardness tester KB 30 S (KB Prüftechnik, Hochdorf-Assenheim, Germany). After the determination of the microhardness, the cube was cut again: first in plane 3 then plane 4 to obtain sample suitable for electron microscopy. Those sample have been grinded and polished again with the above-mentioned procedure to get rid of any damage from cutting and deformations from the determination of the microhardness.

For the examination of the microstructure, a scanning electron microscope FEI—Helios NanoLab 600i equipped with an energy dispersive X-ray (EDS) and circular backscatter (CBS) detector was used. The grain and sub-grain size were measured with the line cutting method according to DIN EN ISO 643 [19].

## 3. Results and Discussion

### 3.1. Evaluation of the Temperature Sensoric

**Figure 5:**
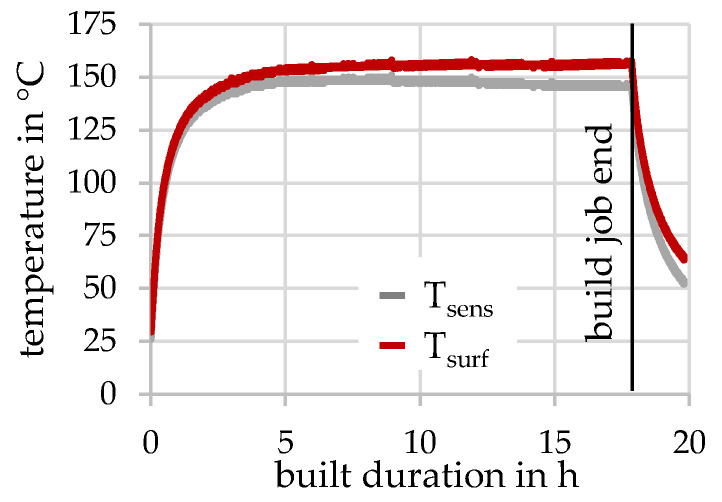
Temperature measured below the built plate and estimated surface temperature.

The evaluation of the built-in temperature sensor T_sens_ gives insights to the current temperature of the specimen, as well as of the surface temperature T_surf_ (see Figure 5). 

A good approximation to the surface temperature is given by the heat transfer through the footprint A of the cube and distance *d* from the temperature sensor below the mounting plate to actual build height. The heat flux can be estimated by the percentual laser ON time *t_ON_* (~72%) during the build job and the mean laser power *P_L,m_* (458 W) needed to expose the areas with each PS.

(1) $$\Delta T=\frac{d\cdot t_{ON}\cdot P_{L,m}\cdot \alpha }{\lambda \cdot A}$$

However, the absorptivity α can be taken from Liu et al. [20] and the temperature dependent conductivity λ from Soylemez et al. [21]. On the one side, it can be seen that it takes almost 5 h until a steady state of around 145–155° is reached. On the other side, it becomes clearly visible that the built height is decisive for the in-situ aging. After the end (~17.8 h) of the build job, the temperature drops quite fast due to cooling effects (e.g., cooling by the gas stream, heat conduction through the powder).

### 3.2. Microstructure

**Figure 6:**
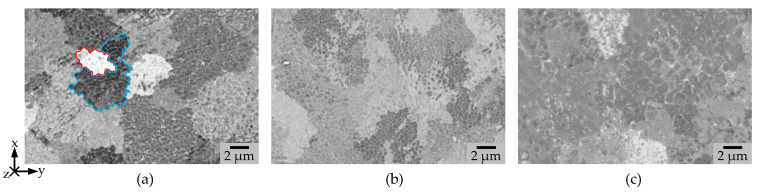
CBS Image taken at a HFW of 25 µm, HV = 10 keV, I = 2.8 nA (**a**) PS 1, (**b**) PS 2, (**c**) PS 3 in the xy plane.

The biggest grain and sub-grain size were obtained by the usage of PS 3 (see Figure 6c).

The use of the single mode laser leads to a significant smaller structure, whereas the grain size between the two used PS with the Gaussian beam profiles can be varied by ~25% (see Table 3).

**Figure 7:**
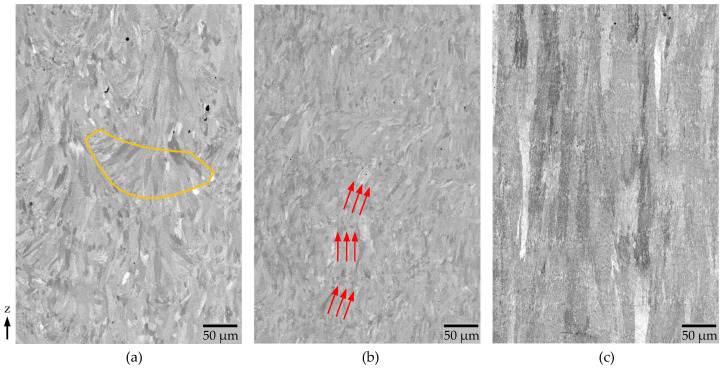
CBS Image taken at a HFH of 500 µm, HV = 10 keV, I = 2.8 nA (**a**) PS 1 melt pool boundaries marked in red, (**b**) PS 2 zig-zag pattern of the grain structure marked in orange, (**c**) PS 3 in the X-Z plane (flipped by 90°).

The gray scale values in Figure 6 of PS 2 and 3 are around the same, whereas for PS 2, the color changes from almost black (Figure 6 region marked in blue) to almost white (Figure 6 region marked in orange). This indicates a lower uniformity of the grain orientation for PS 1 compared to PS 2 and 3. In the CBS images in X-Z Plane (see Figure 7a, marked in orange), the melt pool form can be estimated for PS1. The grains seem to point perpendicular to the weld seams. However, a mixture of short columnar grains and some smaller epitaxial grains at the melt pool border can be found. For PS 3, the grain orientation is strictly oriented into z-direction. The columnar grains have a length of up to almost one millimeter and the weld seam boundaries are almost horizontally oriented. For PS 2, a zig-zag pattern (see Figure 7b, marked in red) similar to previous work by Rasch et al. [22] can be found. 

**Figure 8:**
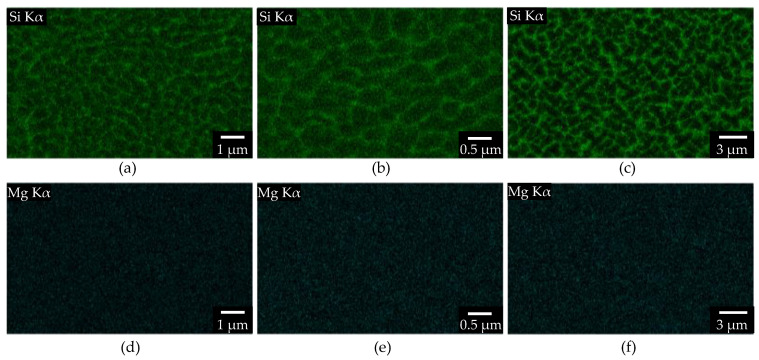
EDS image on various magnification with HV = 10 keV, I = 6.7 nA (**a**,**d**) PS 1, (**b**,**e**) PS 2, (**c**,**f**) PS 3 after 9 h in-situ aging.

These findings regarding the orientation can be attributed to the higher (PS 2 and 3) or lower melt pool overlap (PS 1). The EDS images in the xy 2 plane reveal the distribution of the silicon and magnesium depending on the PS after an in-situ aging of roughly 9 h (see Figure 8).

For all three PS, the silicon is concentrated at the grain boundaries, and magnesium is spread equally over the analyzed cross-section. This indicates, on one hand, that by in-situ aging at ~150 °C, no significant amount of microscale Mg_2_Si secondary precipitations are formed. On the other hand, the impact time of PS 3 is still too short to significantly degenerate the silicon network, despite the huge laser spot and slow scanning speed.

### 3.3. Microhardness

**Figure 9:**
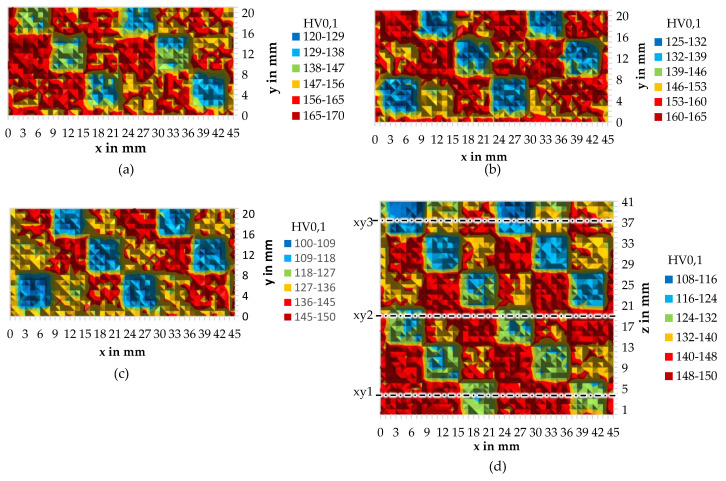
Microhardness HV_0.1_ in different cross sections: (**a**) xy1, (**b**) xy2, (**c**) xy3, and (**d**) xz.

The measured microhardness in cross-section xy 1–3 and xz is shown in Figure 9. The different PS show strong variations in the mechanical properties. The cube’s outlines are still clearly visible despite the long built-time and in-situ heat treatment. The use of PS 2 led to the highest microhardness, whereas PS 3 led to the lowest microhardness. PS 1 is settled in-between but closer to PS 2. Comparing xy 1–3 to xz at the same height levels, the anisotropic mechanical properties become visible due to the columnar grain growth of AlSi10Mg during PBF-LB/M [23]. 

**Figure 10:**
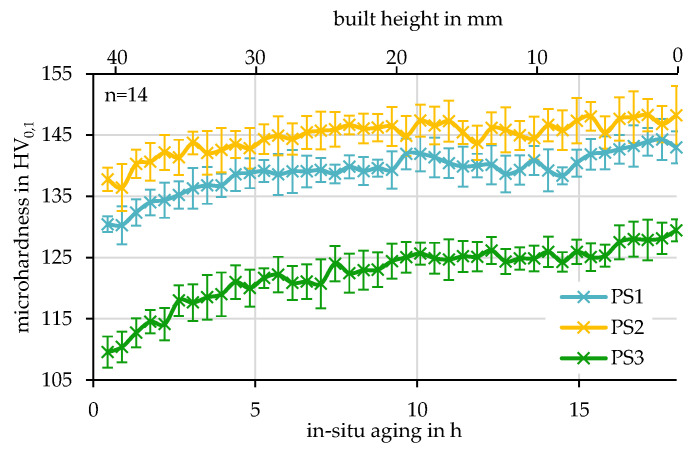
Effect of the in-situ aging time on the microhardness in xz.

The microhardness of PS 1 in xy 3 can be well correlated to the samples without heat treatment examined in [24,25] where similar process parameters have been used. The value obtained by PS 2 in xy3 are comparable to [9] where, beside the same scanning speed, completely different parameters have been used. The properties of PS 3 could not be compared; to the knowledge of the authors, no other experiments with a large spot size are available. In Figure 9d, the effect of in-situ aging described in 3.1 becomes visible. Similar to [25] (180 °C for 6 h~PS 1), the microhardness increases for all used parameter sets, most probably caused by the formation of secondary Mg_2_Si nano-precipitation [26]. In Figure 10, the evolution of the mean microhardness per parameter set due to in-situ aging is evaluated. For PS 3, the percentual increase is the highest. PS 1 and PS 2 share the same increase in HV. 

Quite interestingly, the slope of the hardness evolution changes at around 5–7 h hours of in-situ heat treatment. First, it is steeper rather than much lower. The microhardness in xy 1 reaches even higher values than in xy 2. This is the highest microhardness obtained so far, with an in-situ heat treatment for both parameter sets, PS 1 and PS 2. 

## 4. Conclusions and Outlook

This study aims to bring new insights and contribute to a better understanding on the influence of different parameter sets used within one part. There exists a huge potential of PBF-LB\M to generate parts not only with almost no restriction on the design, but also with the opportunity of tailoring the microstructure locally. The three different parameters set led to different microstructures and correspondingly to different microhardness. It was also found that the parts temperature during the built job causes an in-situ aging which significantly affects the microhardness. As aging is always a function of temperature and time—assuming that the built time cannot be influenced in larger magnitudes—it might be a solution to implement a platform cooling to suppress those effects. This would further support the goal of fabricating parts with locally tailored part properties as the build-height-dependence could be countered.

## Figures and Tables

**Table 1:** Chemical composition of the used powder [16].

	Al	Cu	Fe	Mg	Mn	Ni + Cu	Pb	Si	Sn	Ti	Zn	Total Each
Maximum (wt.%)	Balance	0.05	0.55	0.45	0.45	0.05	0.05	11	0.05	0.05	0.15	0.05
Minimum (wt.%)	Balance	-	-	0.2	-	-	-	9	-	-	-	-
Actual (wt.%)	balance	<0.01	0.12	0.36	<0.01	<0.01	<0.01	9.9	<0.01	<0.01	<0.01	<0.05

**Table 2:** Used parameters.

	Laser Power P_l_ in W	Scanning Speed v_s_ in mm/s	Hatch Distance Δy in µm	Beam Profile	Beam Diameter d_L_ in µm
Parameter set 1	300	1650	130	Gaussian	78
Parameter set 2	360	3250	20	Gaussian	78
Parameter set 3	800	400	350	Top-Hat	650

**Table 3:** Sub-grain size of PS 1–3.

	Sub-Grain Diameter in µm	Standard Deviation in µm
PS 1	0.44	9 × 10^−3^
PS 2	0.34	5 × 10^−3^
PS 3	0.79	3 × 10^−2^
